# Hospital and Institutional News

**Published:** 1914-06-20

**Authors:** 


### HOSPITAL AND INSTITUTIONAL NEWS.

### THE MOUNT VERNON HOSPITAL LITIGATION.

We are informed that the action instituted by the Marquess of Zetland, as President of the above institution, and others against the British Medical Association and members of the late medical staff of the hospital has been arranged. The action, as our readers will remember, was one in which the plaintiffs sought to restrain the defendants from inserting the name of the hospital in the warning notice column of the *British Medical Journal*, consequent upon the resignations of the defendant doctors. The hospital, it is understood, accepts the assurances tendered by the defendants, which it considers satisfactory.

### OUR BED-LIFTER COMPETITION PRIZE SCHEME.

We would remind our readers that June 27 is the last date on which descriptive papers must reach this office in connection with our Second Prize Scheme, full details of which are contained on the third page of the cover. With the object of increasing efficiency on practical points three prizes are offered periodically of Five Guineas, Two Guineas, and a Special Prize, Three Guineas, for a description, in the present case, of bedstead-lifters. The descriptions, which should not exceed 250 words in length, must delineate the best appliance in actual use, or a suggested improvement upon existing articles. The descriptions, which should be accompanied by photographs, or by a rough sketch in the case of suggested improvements, will be submitted to competent judges, and the six best specimens selected from those sent in will be published in The Hospital, when readers will be invited to express their opinions of the respective value of the six designs on special voting forms inserted in The Hospital for the purpose. The competitor who sends the description that secures the greatest number of votes will be awarded the First Prize of five guineas, the one with the Second highest number two guineas, while a Special Prize of three guineas will be awarded to the reader whose voting paper is arranged in accordance with the majority of the opinions expressed. "The Ward Locker," it will be remembered, formed the subject of the prize scheme last month, and we shall deal in turn with every class of institutional requisite and appliance. Any institutional worker who is looking out for a rare type of article would be well advised to send its name to us, so that a prize may be offered for its best design, as in this way the Prize Scheme will be of practical advantage to every institution.

### THE LISTER WARD AT GLASGOW.

In connection with the reconstruction of the Glasgow Royal Infirmary, it has been found that the present position of the Lister ward would be detrimental to the erection of a portion of the projected buildings, and the board of managers therefore decided to remove it. The Glasgow University Court, learning of this decision, have asked to be allowed an opportunity of acquiring the materials of the ward and of the Lister operating theatre, and, after keen discussion and with considerable reluctance on the part of some of the members, the managers have decided to grant the request. The intention is to re-erect the ward and operating theatre in the grounds at Gilmorehill, where it will undoubtedly be a valuable addition to the resources of the University.

### THE EFFECT OF CRITICISM ON BOARDS OF GUARDIANS.

No newspaper can publish a series of signed Reports criticising the standard maintained in the modern hospitals of to-day without learning some valuable lessons from the way in which its frank statements of fact are received. We have been able, from the mass of correspondence which we have received in connection with our Reports, by scientific induction to arrive at the following rule. Few better tests of the capacity and public spirit of institutional boards can be found than the way in which they receive authoritative criticism. What is true of hospitals is no less true of infirmaries and their Boards of Guardians. In our issue of May 30 we recorded the fact that Dr. Fuller, one of the medical inspectors of the Local Government Board, had stated that the sick wards in the Bath Workhouse were totally unfit for the accommodation of the sick; and, further, that Dr. Fuller had stated that the present building was such that he would not like to carry out even a minor operation within it. We also recorded that the Bath Guardians, in the face of this grave and authoritative criticism, had decided, by thirty votes to twenty, to shelve the question of building a new hospital for the workhouse for one year. Our readers will since have read the letters of the chaplain, the Rev. Humphrey Hirst, and Dr. Samuel Craddock, the late medical officer, indignantly repudiating certain reports which we quoted in the paragraph referred to.

### THE IMPRESSION CREATED BY THE BATH GUARDIANS.

In the meantime, as usual, letters not intended for publication continue to arrive, but in the public interest it is necessary to record the point which prompts them. It is this, that, put shortly, the Bath Guardians are so sore at the facts which we recorded and the criticism which briefly followed them, that all chance of a new infirmary has been spoiled for the present. Previous experience of public authorities indicted for errors which have lasted a long time, like the structure of the sick wards of the Bath Workhouse, has taught us that ancient defects become cherished in the course of years, and that it may well be true—astonishing as it is to men of public spirit—that the Guardians may nurse their collectively bad conscience by determining to postpone reform for as long as they can. Were no other facts before us, were Dr. Fuller's and Dr. Curd's statements not available, the fact that this impression is now rife in Bath would lead us from our experience to condemn the Bath Guardians as administratively behind the times. For does not the above impression represent the plaintive attitude of those but half-awakened from long slumber, and really come to this, "If you venture to criticise me, I will stand on my dignity and refuse to listen"? This is all very well in schoolrooms, but it is unworthy of the city of Bath, and we hope that the Guardians will take an early opportunity of removing this disastrous impression from the inhabitants by assuring them that proper plans for new sick wards will be prepared without delay.

### A VETERAN MEDICAL JOURNALIST.

As a good many people suspected from the first, it turns out that the anonymous "F.R.C.S." whose letter to the *Times* (alluded to in THE HOSPITAL, June 13, p. 308) created so much excitement and provoked so many angry replies, is really Mr. Brudenell Carter, the celebrated ophthalmic surgeon. Mr. Carter, who has retired from practice, first came into prominence during the Crimean war, sixty years ago. His letters from the front, where he was employed as a civil surgeon with the troops, attracted so much attention and encomium that on his return he was invited to join the staff of the *Times*. Throughout a long institutional career, and in spite of the claims of private practice, Mr. Carter wrote regularly for our premier newspaper for more than half a century; and his recent letter amply proves that his pen has not even now lost its cunning. Dowered with a singular lucidity, and a very pleasing and elegant style of English composition, Mr. Brudenell Carter's work has always stood for the highest ideals of British journalism; and it always commanded to a peculiar degree the admiration of his colleagues in Printing House Square and Fleet Street. With a record thus unique behind him, it is strange that Mr. Carter's many and varied claims to distinction have never been recognised officially. Crimean veterans of far less merit have been loaded with honours; we have none too many of them with us now to let any of them remain neglected.

### CHARING CROSS HOSPITAL HISTORY.

THE contagion of well-doing seems to be spreading rapidly among institutional historians. This week another well-known hospital is the subject of a publisher's announcement; for Mr. Murray has almost ready for publication a "Historical Account of Charing Cross Hospital and Medical School" by Dr. William Hunter, now and for some years Dean of the Medical School there. We learn that many old prints and plans are reproduced, illustrating the growth of London and the districts served by Charing Cross Hospital. A synopsis is also being included of the origin of the other London schools and hospitals, tracing the progress of medical science in London. This latter section follows the example set by Mr. Peachey, whose history of St. George's is still appearing in parts; for the latter author devoted an important portion of his history to a sketch of the institutional world as it existed in the first half of the seventeenth century, the period when so many of the famous London charities were founded. We hope to have an opportunity of reviewing this book, which will be of great interest to all institutional workers, as soon as it is published.

### TUBERCULOSIS PAVILIONS AT ISOLATION HOSPITALS.

AFTER a lengthy discussion the health committee of the Taunton Town Council has decided against the County Council scheme for the erection of a pavilion for the treatment of tuberculous cases at the Taunton Isolation Hospital, although it is willing to join in a conference to discuss the matter. The clerk to the Isolation Hospital Joint Committee, Mr. W. F. B. Dawe, in the course of a correspondence with the health committee, pointed out that a similar arrangement to that proposed above had been carried out with the Local Government Board, in the grounds of Shepton Mallet Hospital. The saving to the ratepayers of the expense of an entirely separate staff—which would be made possible by "proper provision being made" so that "no risk would be incurred"—was an important arrangement. The undertakings suggested were that the County Council would provide the necessary wards on lands given by the Hospital Committee, while the County Council would pay 21s. per week for each case of tuberculosis to the Hospital Committee, and provide such payment for at least fifteen cases at any time. As a condition of such payments the Hospital Committee would give up sufficient land; "arrange for the general administration of the tuberculosis ward as part of their general hospital," provided no extra building is required; to provide and pay for the additional staff required; to pay all additions to the salaries of the existing staff; and "to feed and maintain the cases to the satisfaction of the county tuberculosis hospital." The arrangement, in its financial aspects, was to be open to revision after six months, and would continue during the currency of any loan raised by the County Council. Finally, the County Council were to agree to pay the hospital, in addition to the above sums, £30 per annum for the use of land and administration block.

### A QUESTION OF DUAL CONTROL.

WE have thought it well to summarise the main features of this agreement, not because one isolation hospital has decided to accept it, but mainly as an indication of the manner in which the County Councils are endeavouring to meet their Parliamentary obligations at the present time. Alderman Whittingham, chairman of the Hospital Joint Committee, declared that Dr. Savage, medical officer of health, assured him there was no danger whatever in having the two hospitals in conjunction. Dr. Henry J. Alford, M.D., Taunton Borough medical officer, asserted that he had been ignored by Dr. Savage in the matter and criticised the scheme from the administrative point of view, finally condemning it as "preposterous" both financially and on account of its risk. In view of the suggestion that the County Council should make good any deficiency, Dr. Alford's animadversion on the financial side of the scheme appears to lack force. But administratively what can be said of a plan under which two contiguous institutions dealing with different types of infectious patients are to be run by one staff, and in which the diet and treatment of one set of patients has to be controlled by an outside official, the county tuberculosis officer, who at the same time will have a staff largely under the orders of another, resident, medical man?

### THE DEAN OF YORK RESIGNS HIS CHAIRMANSHIP.

The chairman of York County Hospital, the Very Rev. Purey-Cust, Dean of York, has tendered his resignation of this post after having held it for thirty-four years. The Dean's experience of hospital work extends, however, over an even longer period. During the fourteen years when he was at Reading he was a member of the committee of the Royal Berkshire Hospital, and when he passed on to Aylesbury he held the post of chaplain, after all of which his chairmanship of the York County Hospital began. As a matter of fact it is over fifty years—or, to be precise, for fifty-four years—that he has been engaged in institutional work, and now that he is so greatly advanced in years and that his faculties, particularly his hearing, have become impaired, no one will grudge him his well-earned retirement to which his services clearly entitled him several years ago. It is characteristic of the attitude of the committee of York County Hospital, and of the regard which they have for the value of the Dean's work, that they have invited him to accept the presidency of their institution, an office which carries with it the *ex-officio* membership of the house committee. The work, then, of maintaining the County Hospital at the level of the modern standard devolves primarily on the new chairman, Mr. W. F. H. Thomson. The task is an opportunity for an able man.

### THE POLICY OF SHUNNING PUBLICITY.

At the monthly meeting of the executive committee of the General Infirmary, Worcester, over which the Rev. Canon Longhurst presided, it was reported that two probationers had died of typhoid fever. Thereupon the Dean of Worcester questioned the advisability of allowing this fact to be made public, but it was represented to him that, having regard to the feeling outside, it would be in the interest of the infirmary that something should be said about it. The Chairman remarked that even the best managed institutions were liable to such distressing circumstances, but that did not relieve them of their sense of responsibility for doing all they could to mitigate the consequences. The Clerk explained that it was believed the disease was contracted from a child admitted as a case of appendicitis. It is very lucky for the infirmary and the Dean that this decision was arrived at. The policy of shunning publicity is always fatal and recoils on the heads of those who enforce it. Facts must be faced; gossip is the result of suppression.

### SECRETARIAL CHANGES AT WORCESTER INFIRMARY.

A letter from Mr. W. Stallard, the secretary of the Worcester General Infirmary, which was read at the meeting on Monday last, stated that fresh work had been thrown upon the secretary which must necessarily be done at the infirmary. He asked the committee to consider the question of transferring all the office work to the infirmary, and suggested that on the 24th inst. he should cease to be the paid secretary, and that Mr. E. J. Holland, assistant secretary, be appointed in his stead at a salary of £125 per annum, being allowed at the same time to retain the secretaryship of the Ophthalmic Hospital. Mr. Holland would have the assistance of himself and his assistants. After next March Mr. Holland's whole time might be given to the infirmary, and he would probably then require the help of a junior. This arrangement would result in a saving to the infirmary of £50 per annum. There was much discussion on this suggestion, which the house committee was disposed to accept. The Chairman, however, said that he regarded the resignation as a serious matter for the infirmary, and expressed his feeling that before they took a step of that kind they should consider the matter carefully. He saw there was a good deal going on outside which was not in the interests of the infirmary. He appealed to Mr. Stallard to defer his action for three months, so as to give the committee time to consider further the proposal. This suggestion was supported by Mr. Harris, who took the opportunity of expressing his appreciation of Mr. Stallard's unfailing courtesy and kindness towards the committee, and to himself especially. So the matter has been postponed. It is rather interesting to note, however, that the secretarial duties are said to have increased, as this proves how futile is the policy of closing beds in the hope of saving expense, since the cost of administration, the most important factor in hospital accounts, remains unaffected by it. Out of their own mouth this judgment is recorded at Worcester.

### INSTITUTIONAL EDUCATION FOR THE MENTALLY DEFECTIVE.

The clauses in the Mental Deficiency Act in which many institutions coming within its purview are most interested are those which concern the training of all educable mentally defective children, for which purpose the education authorities are compelled to provide facilities within their areas, by special classes or residential schools. Manual training necessarily forms an important part of this instruction, and the place of residential schools is taken in some places by institutions for the reception of improvable cases, of which the various industrial colonies (some of which devote a part or all their accommodation to this class of case) are examples. Among institutions for the training of improvables, that at Starcross, near Exeter, is interesting, for it helps to supply the need of the western counties. It is no doubt partly because its committee hope to be able to co-operate with the education authorities that they have amended the title from that of mental hospital to simply the Western Counties Institution. Existing voluntary institutions for the feeble-minded like this one, which were worked under the Idiots Act of 1886 till its repeal on the passing of the new statute, originally feared that the Mental Deficiency Act would compass the end of their career of usefulness. The strong Parliamentary Committee formed on their behalf, and the growing perception of the huge cost entailed by the scheme even were all existing suitable accommodation utilised, managed to secure the embodiment of important alterations, but even at present committees of voluntary institutions are feeling restricted in their action, while the large amount of statistical work imposed on them, including certificates and reports, is still causing them trouble. Mr. Ernest W. Locke, the secretary and superintendent, has therefore had the responsibility on his shoulders of arranging a suitable form of co-operation with the education authorities, but he can have the—perhaps to him at present doubtful—compensation of seeing the type of voluntary institution to which his institution belongs taking on a new official importance and a more publicly extended prestige.

### THE DOCTORS' WIVES ASSOCIATION.

The preliminaries for beginning the work of this Association are now practically complete, and we are happy to be able to inform those interested that the doctors' wives' co-operative scheme will be sufficiently advanced to enable the work to commence from July next. Every member who has joined will receive full particulars of the conditions which will govern the use of this Association by doctors' wives.

### THE INSECURITY OF THE MEDICAL OFFICER OF HEALTH.

Sir Philip Magnus, M.P., and Dr. Addison, M.P., introduced last week to the Chancellor of the Exchequer a deputation arranged by the British Medical Association and the Society of Medical Officers of Health. Its object was to request greater security of tenure for medical officers, and for the extension of superannuation. In saying that their case had only to be stated to carry conviction, Mr. Lloyd George remarked that medical officers of health were the pivot on which health administration turned. An essential part, if not an essential preliminary to any further legislation dealing with the housing question, was sufficient security of tenure to give independence to this branch of the profession. Executive officers should be able to report without fear or favour on the actual condition of their areas. The principle of superannuation was one with which he agreed, and he stated that Mr. Herbert Samuel, the President of the Local Government Board, was now investigating the whole question. Our readers will not have forgotten the recent disclosures in Dublin and the statements made to the effect that some of the principal owners of slum property were the municipal authorities of Dublin. The present condition of affairs can be reduced to absurdity by drawing the unexaggerated parallel that medical officers of health are in the same position as factory inspectors would be supposing that the right of appointing and dismissing the inspectors were vested in the factory owners. The wonder is that medical officers of health show so much public spirit and integrity as they do, but to believe that there is the smallest general possibility of them being able to report without fear or favour at the present time is unfortunately preposterous. What does Mr. Herbert Samuel propose to do?

### HOSPITAL RISKS OF INFECTION.

Hamel of Berlin has made a wide investigation of the above in connection with tubercle, questioning over 2,000 doctors and 13,000 nursing staff and attendants, with the general result that the lower the rank of the official, and consequently perhaps the more prolonged and close the contact with the patient, the greater the risk. Head physicians suffered six times as seldom as assistant ones, nurses generally more than doctors, ward-maids more than the nurses and twice as often as the hospital porters. In hospitals of old design the incidence of infection was as much as twice that in new, up-to-date institutions. On the other hand, the proportion of staff to beds seemed to make no difference. There is no doubt that to be constantly in even the best-planned hospital, even in these days of asepsis, is not a very healthy thing. Tuberculous disease was thirty per cent. more frequent in the assistant medical staff than amongst practitioners generally. However, the matter of age has to be considered here; there would be more of the former in that vulnerable (to consumption) period of life, namely the third decade. It is not often that one can suggest anything further in a German investigation, but we might note that our recent query as to the morbidity of hospital secretaries is not answered by the above.

### NEW RESEARCH LABORATORIES AT THE INFANTS' HOSPITAL.

It is a great tribute to the hold on the affections of public men which the voluntary system maintains that the new research laboratories at the Infants' Hospital, Vincent Square, which were opened by the Duchess of Albany last week, should have been the gift of one man—namely, Mr. Robert Mond. As the hospital was built only in 1907 it may consider itself fortunate in having attracted such constant and active enthusiasm, and the new laboratories have a special interest from the fact of our general ignorance of diet. If Dr. Ralph Vincent and his staff of women workers, who are six in number, succeed not merely in elucidating some obscure problems of infantile diseases, but also in drawing general conclusions from their investigation of diet, the world at large will have a very great deal to be grateful for. We do not well know where scientifically nourishment ends and stimulus begins. The whole question of what really constitutes diet has to be worked out, without reference either to rabid meat-eaters or sentimental vegetarians. If time can be found for these problems in the lower ground floor of the new buildings, which constitutes the research laboratory, then Mr. Mond will have built better perhaps than he knew. The rest of the block is given up to an out-patient department on the ground floor, and above to separate bedrooms for nurses. The cost of the new block is £12,000, of which £5,000 was spent on the laboratory and its equipment.

### THE AMATEUR ALIENIST.

No doubt there are some men in general practice who have a special gift in regard to the efficient treatment of the insane; but only too often it is to be observed that, as a result of tentative efforts at treatment by those inadequately instructed in the proper care of the mentally deranged, recovery has been deferred rather than brought nearer. The majority of people who would not venture to assert their ability to discuss the treatment of such conditions as fractures, pneumonia, meningitis, and so on, quite confidently set up their opinion when insanity is under discussion, not realising that only practical experience—and that, too, of a prolonged character—is necessary before anything like a reasoned opinion can be given in matters pertaining to morbid psychological states. In the treatment of insanity too much is already left in respect of supervision to the amateur alienist.

### REMINISCENCES OF THE SEPTIC EPOCH.

A well-known and much respected Glasgow practitioner, Dr. David Pride, has published a volume entitled "Reminiscences of a Country Doctor, 1840-1914," which is being favourably commented on in the Press. Most of the veterans in the medical profession omit to chronicle on paper their recollections of bygone days, and it is much to the doctor's credit that he has made himself an exception to the rule. Dr. Pride is a J.P. for Renfrewshire, and his professional doctorate was obtained in Glasgow more than half a century ago. Moreover, his memory is full and accurate, so his memoirs are naturally interesting reading for Glaswegians. In a very few years the pre-Listerians will be so scanty a band that every one of them will be justified in publishing reminiscences of the septic epoch, "lest we forget." (See Dr. Wrench's "Lister's Teaching.")

### GUARDIANS' SUBSCRIPTIONS TO VOLUNTARY INSTITUTIONS.

There are signs of a movement by Metropolitan Boards of Guardians more adequately to appreciate the great assistance to their work rendered by the hospitals. Since the Insurance Act came into force the number of persons requiring aid from these and kindred societies has largely increased, with little or no additional financial benefit to the organisations concerned. The Southwark Board of Guardians have decided to double their annual subscription to the General Lying-in Hospital, York Road, by raising it from ten to twenty guineas. In the last six months 102 patients from Southwark were admitted to the hospital, eleven cases being sent by the relieving officers. The Wandsworth Board of Guardians have increased their subscription to the Southwark Diocesan Association for the Care of Friendless Girls to fifty guineas, a useful organisation which assists the South London Guardians in their work.

### A HOSPITAL SECRETARY AS ARCHITECT.

The Newbury District Hospital's two new private wards for paying patients are to be opened on July 6. Among the interesting points about them is the fact that they are stated to have been designed by Mr. J. A. H. C. Borgnis, the hon. secretary. The exterior is in the Georgian style, and, though there is an entrance from the main building for the use of the staff, the new wards are self-contained, and possess a separate entrance for patients and visitors, and also an inclined pathway up which an ambulance can be wheeled. A waiting-room for patients' friends where tea may be served to them is also placed on the ground floor. The dimensions of the two pay-wards are respectively 18 feet by 15 feet 3 inches, and 14 feet 6 inches by 12 feet 6 inches. On the other side of the corridor a bath-room, small kitchen, and a sink-room fitted with forced water-pressure appliances for cleansing macintoshes, pans, and bottles are placed. The new wards form a memorial to the late Dr. Kerby, formerly one of the medical officers at Newbury District Hospital, and his widow has given £200 towards the expense of building and furnishing, which has amounted to £650. Once the wards are paid for it is hoped that they will be self-supporting, and indeed the sanguine statement has been made that "they will prove a source of income." We hope that this is not the official view, and that Mr. Borgnis and Mr. H. J. Vollar, the member of the committee perhaps most keen on the scheme, have not endorsed it. It may very easily not prove correct, for two things have to be remembered.

### THE OBJECT OF THE PAY-WARD.

The first is that for pay-wards to produce a profit the fees charged will have to be on the same scale as those usual in a nursing home, and therefore beyond the reach of the class of patients which hospital pay-wards are designed to benefit. Secondly, such a fee will inevitably bring the hospital into competition with the nursing homes of the district, which would lead to an unnecessary and harmful condition of affairs; or if there be no nursing-home close at hand one will soon be started, when this difficulty will occur; and what will the doctors say to attending, in the hospital, patients who can afford nursing-home fees? All experience goes to show that £2 2s. a week is the maximum figure to be charged in pay-wards attached to our existing hospitals. Under this condition they meet a real need, and avoid the difficulties mentioned above; but no profit or source of income can arise from them at this figure.

### PRINCE ALEXANDER OF TECK'S SUCCESSOR.

UNUSUAL interest was given to the second annual meeting of the Middlesex Hospital Ladies' Work Society, which was presided over by Prince Alexander of Teck last week, by the announcement that he had, in the words of Mr. Arthur Stanley, found a substitute in Prince Alexander of Battenberg. Again the hope was expressed that the Prince would be able once more to resume the work of the hospital on his return from his period of office in Canada.

### THE NATIONAL HEALTH LABORATORY.

PROFESSOR BENJAMIN MOORE, well known in Liverpool and elsewhere as an advocate of a State Medical Service, has received a Government appointment in connection with the National Health Laboratory, which is to be opened in October next at Hampstead. The laboratory—for which £15,000 has been found out of the £60,000 available for research work from the penny from each of the fourteen million insured patients—will investigate with its staff of experts the causes of disease attributable to industrial occupations. Thus a specially equipped and central department will undertake the work for which small " departments " associated with the Local Government Board and the Board of Trade have been more or less in haphazard fashion responsible. The laboratory work is expected to be divided into three sections, bacteriology, experimental psychology, and applied physiology and hygiene. It is to this last that Professor Moore will be attached with Dr. Leonard Hill. Educated at Belfast, Leipsic, and Yale University, Mr. Moore held a lectureship in physiology at Charing Cross Medical School previous to his appointment as Johnston Professor of Bio-chemistry at Liverpool, which he now vacates on his transference to the Government post in London. Among his works for popular consumption " The Dawn of the Health Age " has been eagerly read by laymen in the prevailing fashion for semi-medical, sociological, and scientific reading. A section of the medical profession, which was in favour of the arguments advanced, also read it with appreciation and pleasure.

### A MEDICAL STATISTICIAN.

DR. JOHN BROWNLEE, physician superintendent of Ruchill Hospital, N.B., has been appointed medical statistician under the Government Medical Research Committee. Graduating at Glasgow University in 1894, he has had appointments at the Victoria Infirmary, the Fever Hospital at Belvedere, and as superintendent of the Fever Hospital, Kennedy Street, and of the Smallpox Hospital. He then became assistant to the medical officer of health for Glasgow, till he received the senior post in Guernsey, and was appointed to Ruchill Hospital in 1909. Dr. Brownlee was awarded last year the Macdougall Brisbane prize for statistics by the Royal Society of Edinburgh.

### THE ISLINGTON TUBERCULOSIS SCHEME.

THE borough council of Islington—and the district forms the largest borough in London—have just accepted a scheme for the control of tuberculosis in that district. Subject to the approval of the Local Government Board this scheme is expected to be in working order by October next. The plan suggested follows closely the scheme now at work in the neighbouring borough of Shoreditch, which has been described in these columns. Use is to be made of existing hospitals; two dispensaries are to be started—one at the Great Northern Central Hospital and one at the Royal Hospital for Diseases of the Chest in the City Road. The borough will be divided into north and south portions, and a tuberculosis officer will be appointed in each division. As this borough is the largest in London it remains to be seen if two officers only can cope with the work. This scheme was not passed without a considerable amount of acrimonious discussion, and we understand that there were those in favour of a municipal dispensary. The latter is always more popular with medical officers of health, as capable of being better controlled. The Chest Hospital in the City Road has had already nearly two years' experience in dealing with the tuberculosis problem in Shoreditch, and will doubtless be able to cope with the more than double amount of work which will have to be done in its tuberculosis dispensary when South Islington comes into its sphere. Consultants are to be appointed, we understand, at each hospital to assist the tuberculosis officers; but it is to be hoped that the authorities will profit by the experience of other bodies and not seek to attract inferior or junior men by offering inadequate salaries.

### THIS WEEK'S DRUG MARKET.

A FAIR all-round business has been done, but fluctuations in value have been few and for the most part unimportant. Quinine has again been in steady demand, the opinion being held in some quarters that makers will shortly raise their prices; an advance in due course seems inevitable, but whether or not it will take place in the near future remains to be seen. There is little change in quotations for cod-liver oil, but the tone of the market seems somewhat weaker, and it would be possible to make purchases at prices slightly lower than those recently ruling. Essence of lemon is dearer in consequence of an understanding between producers, but there is some doubt as to whether the advanced prices can be maintained. Citric acid continues scarce and in good demand, and there seems to be little doubt that the high prices will be maintained for the present. Tartaric acid is also in good request. Nux vomica is scarce and very dear, and consequently makers of strychnine have raised their prices. Ergot of rye tends upwards in price. Menthol is unchanged. At the recent public auction of drugs in Mincing Lane slightly lower prices were accepted for Jamaica sarsaparilla, and cardamoms were dearer; but on the whole price changes were few and unimportant.

